# Supplementary material for: Concentration Polarization Enabled Reactive Coating of Nanofiltration Membranes with Zwitterionic Hydrogel
Source: Membranes (Basel). 2021 Mar 9;11(3):187. doi: 10.3390/membranes11030187 (PMC7999987; doi:10.3390/membranes11030187)
Supplement: Supplementary file 1 [file membranes-11-00187-s001.pdf]

Article

Concentration polarization enabled reactive coating of nanofiltration membranes with zwitterionic hydrogel

Patrick May, Soraya Laghmari, Mathias Ulbricht

Lehrstuhl für Technische Chemie II, Universität Duisburg-Essen, 45141 Essen, Germany

Section S1. NMR spectrum of P(SBMA-co-MAHEMA) and calculation of composition

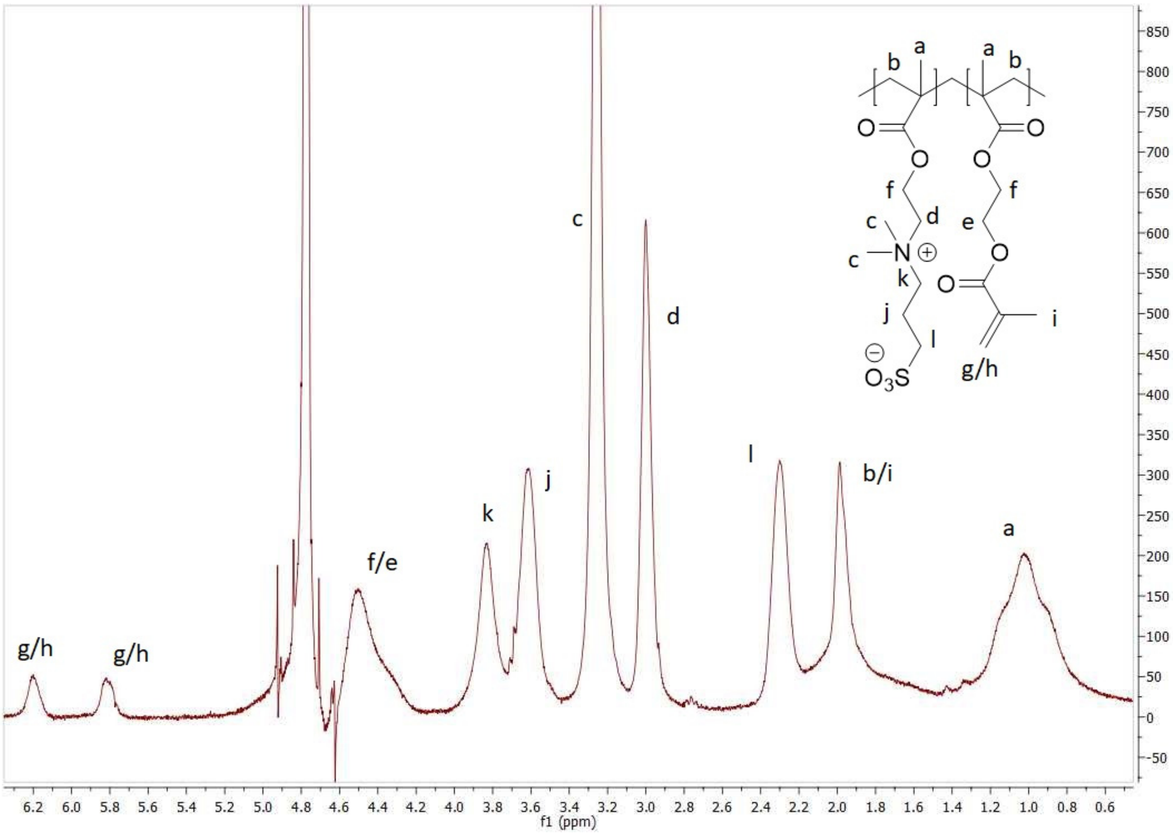

$$zwitterionic\ units[\%] = \frac{\int \frac{c}{6}}{\int \frac{a}{3}} \tag{1}$$

$$methacrylate\ units[\%] = \frac{\int g}{\int \frac{a}{3}} \tag{2}$$

Figure S1. ^{1}H-NMR spectrum of P(SBMA-co-MAHEMA) in D_2O with assignment of peaks to molecular structure as well as formulae used for calculation of functional group content.

Section S2. Molecular weight calculation

Calculation of MW of P(SBMA-co-MAHEMA) from MW of P(DMAEMA-co-HEMA) determined by SEC was done with the following equation:

$$M_{n,functionalized} = M_{n,0} * \frac{\overline{MW}_{side-chain}\ (PSBMA-co-MAHEA)}{\overline{MW}_{side-chain}\ (PDMAEMA-co-HEMA)} \quad (3)$$

Where $M_{n,functionalized}$ is the MW of P(SBMA-co-MAHEMA), $M_{n,0}$ is the MW of P(DMAEMA-co-HEMA) and $\overline{MW}_{side-chain}$ is the average molar masses of P(SBMA-co-MAHEMA) or P(DMAEMA-co-HEMA) side groups, considering the composition determined by ^{1}H NMR spectroscopy.

Section S3. Free bulk gelation conditions

Table S1. Free bulk gelation conditions

Polymer concentration [wt.%]	APS:Acrylate ratio	APS:TEMED ratio
**4**	1:2	1:8
**5**	1:10	1:8
**5**	1:5	1:8
**5**	1:2	1:8
**7.5**	1:10	1:8
**7.5**	1:5	1:8
**7.5**	1:2	1:8
**10**	1:10	1:8
**10**	1:5	1:8
**10**	1:2	1:8

Section S4. Mathematical derivation of equations for calculation of development of concentration polarization during dead-filtration of copolymer

Calculation of CP was based on the assumptions of the film model. Only the zwitterionic cross-linkable copolymer, as the component of key importance for the formation of a hydrogel, was considered. However, due to dead-end mode, CP is dynamic and, hence, the mass balance for transport of copolymer in the boundary layer was calculated in an iterative manner for short subsequent time intervals (1 s).

Convective mass transport into the boundary layer

First, convection causes mass transports towards the membrane surface, expressed as:

$$m_c(t_n) = V(t_n) * c_f(t_n) * R_i \tag{4}$$

with $m_c(t_n)$ as convectively transported mass in interval n (number of intervals with constant duration time of 1 s), $V(t_n)$ as permeated volume through the membrane in interval n, c_f as copolymer feed concentration and R_i as rejection of component i (for the copolymer and a NF membrane R = 1).

Resulting <u>average concentration in the boundary layer</u> at the membrane surface was then calculated by:

$$c_{bl}(t_n) = \frac{m_c(t_n)}{A*\delta} + c_f(t_n) \tag{5}$$

with $c_{bl}(t_n)$ as the average copolymer concentration in the boundary layer in interval n, A as effective membrane area and δ as boundary layer thickness estimated from independent filtration experiments (see section 2.6.2 of main paper).

Back diffusion

Mass transport away from the membrane surface needs to be also considered for proper estimation of concentration, the following equation gives average concentration $\bar{c}_{bl}$ for time interval t_n:

$$\bar{c}_{bl}(t_n) = \frac{c_{bl}(t_n) + c_{bl}(t_{n-1})}{2} \tag{6}$$

Mass transported back toward the bulk feed by diffusion $m_D(t_n)$ is driven by the concentration gradient according to Fick's law:

$$m_D(t_n) = \frac{\overline{c_{bl}}(t_n) - c_f(t_n)}{\delta} * D_p \tag{7}$$

with D_p as the diffusion coefficient of the copolymer.

Viscosity dependent diffusion coefficient of the copolymer P(SBMA-co-MAMMA) was determined via Stokes-Einstein equation:

$$D_P(t_n) = \frac{k_B T}{6\pi\eta(t_n) r} \tag{8}$$

With k_b as Boltzmann constant, T as room temperature, r as hydrodynamic radius obtained from DLS measurements (see section 2.3.3 of main paper) and $\eta(t_n)$ as time dependent dynamic viscosity of the copolymer solution.

Concentration dependence of viscosity of the copolymer solution was derived from rheological experiments (see section 2.3.4 of main paper) and calculated for average concentration at the membrane surface:

$$\eta(t_n) = \bar{c}_{bl}(t_n) * \frac{d\eta}{dc} \tag{9}$$

with $\frac{d\eta}{dc}$ as the change of viscosity in dependence of copolymer concentration.

Net transported mass into the boundary and corrected concentration in the boundary layer

Net transported mass m_{net} is obtained by subtraction of mass transported by back diffusion from that transported by convection, resulting in the corrected concentration in the boundary layer $c_{bl,c}$, given by:

$$m_{net}(t_n) = m_c(t_n) - m_D(t_n) \tag{10}$$

$$c_{bl,c}(t_n) = \frac{m_{net}(t_n)}{A * \delta} + c_f(t_n) \tag{11}$$

Next Iteration

Finally, iteration for next time intervals is performed with, using the formula for corrected concentration in the boundary layer:

$$c_{bl,c}(t_{n+1}) = \frac{m_{net}(t_{n+1})}{A * \delta} + c_f(t_{n+1}) \tag{12}$$

$$c_f(t_{n+1}) = c_f(t_n) + \frac{m_D(t_{n+1})}{V(t_{n+1})} \tag{13}$$

Note, that $V_f(t_n)$ as feed volume permeated through the membrane in the respective time interval n changes with filtration time; i.e. the flux as function of time is used as input parameter.

Linear concentration gradient in boundary layer

However, the above derived calculations is valid only for homogenous distribution of net mass in the volume of the boundary layer, i.e. the average concentration in the boundary layer. Thus, more realistic extent of CP can be derived by assuming a linear concentration gradient in boundary layer for same transported net mass (Scheme S1). The corresponding concentration at membrane surface c_m is derived from:

$$c_m(t_n) = 2(\frac{m_{net}(t_n)}{A*\delta} + \ c_f(t_n)) \tag{14}$$

The iteration for each next time interval is performed as described above, using:

$$c_m(t_{n+1}) = 2(\frac{m_{net}(t_{n+1})}{A*\delta} + \ c_f(t_{n+1})) \tag{15}$$

$$c_f(t_{n+1}) = c_f(t_n) + \frac{m_D(t_{n+1})}{V(t_{n+1})} \tag{16}$$

The estimation of CP was performed to evaluate membrane modifications. Finally, the comparison between real and estimated outcome allowed to judge validity of proposed model.

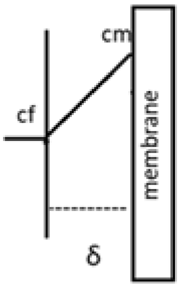

Scheme S1. Linear increase in concentration polarization – used assumption in this work.

Section S4. Storage modulus of bulk hydrogels

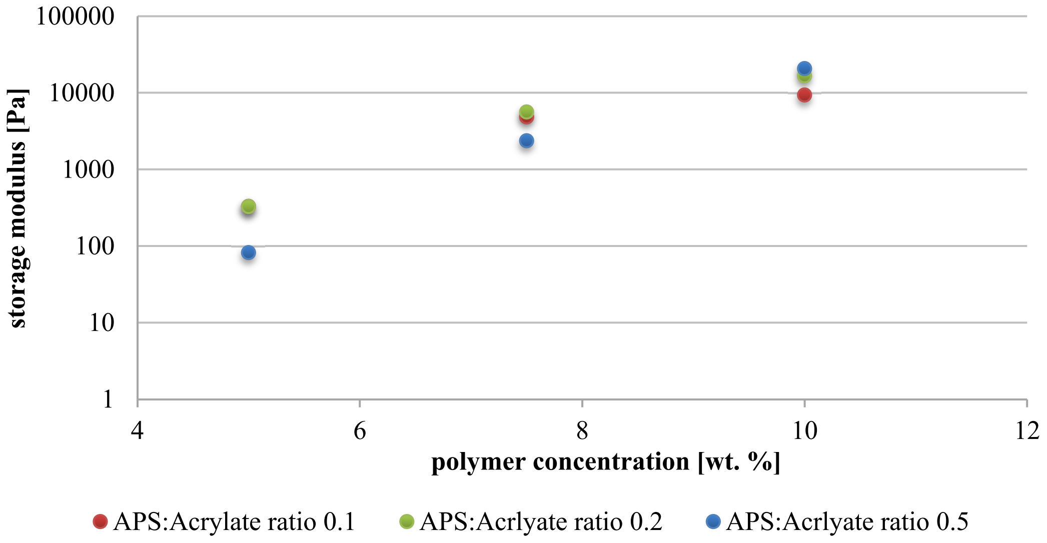

Figure S2. Influence of polymer concentration on storage modulus of hydrogels obtained by in situ cross-linking in bulk.

Section S5. Characteristics of base membrane used for modification

Table S2. Characteristics of NF 270 membrane

Characteristics	*Exp.*	*Lit.*
NaCl rejection [%]	37.7 ± 7.8	50-52
Na_2SO_4 rejection [%]	89.7 ± 2.8	90-95
TEMED rejection [%]	36.5 ± 9.5	-
APS rejection [%]	75.9 ± 3.1	-
zwitterionic polymer rejection [%]	100	-
pure water permeance [L/hm²bar]	11.3 ± 1.0	17.0 ± 0.8
contact angle [°]	32.5 ± 1.3	29-55

Section S6. Flux during modification

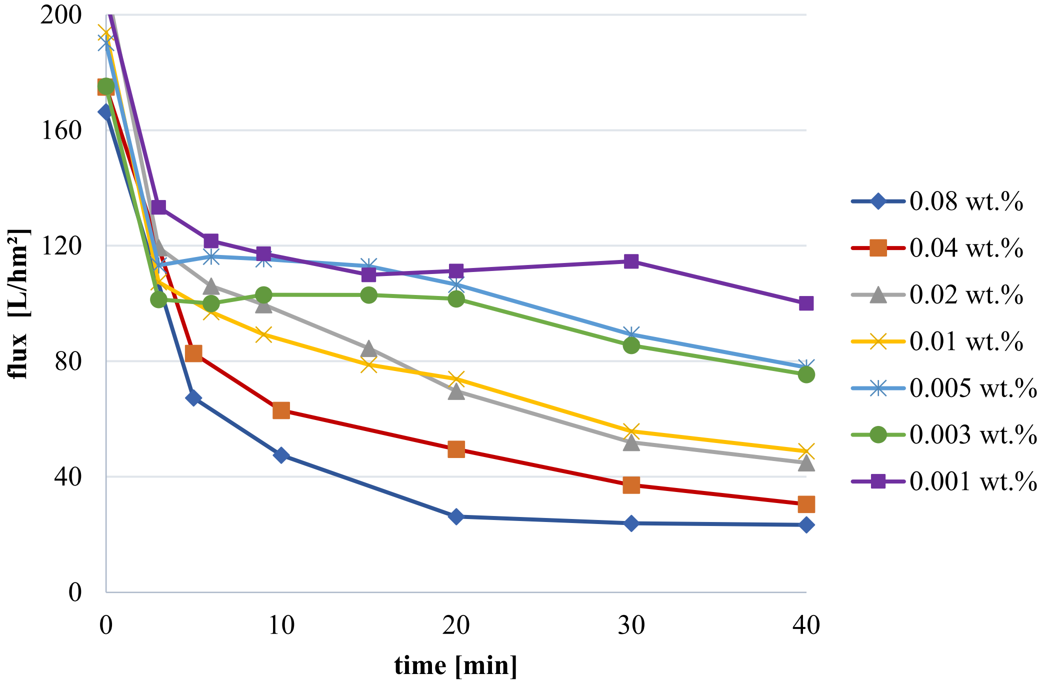

Figure S3. Change of flux during filtration / modification of NF270 membranes with reactive solutions containing different copolymer concentration (cf. Table S1).
